# Efficacy and Impact of Peer-Led Education for Persons with Tuberculosis in Kampala, Uganda: A Pre-Post Implementation Study

**DOI:** 10.21203/rs.3.rs-3956897/v1

**Published:** 2024-02-16

**Authors:** Anna Baker, Amanda J. Gupta, Leah Nanziri, Joseph M. Ggita, Raul U. Hernandez-Ramirez, Sheela V. Shenoi, Irene Ayakaka, Mari Armstrong-Hough, Achilles Katamba, J. Lucian Davis

**Affiliations:** Yale School of Public Health; Yale School of Public Health; Uganda Tuberculosis Implementation Research Consortium; Uganda Tuberculosis Implementation Research Consortium; Yale School of Public Health; Yale School of Medicine; Uganda Tuberculosis Implementation Research Consortium; New York University School of Global Public Health; Makerere University College of Health Sciences; Yale School of Public Health

**Keywords:** tuberculosis, health education, counseling, implementation science, Uganda

## Abstract

**Introduction:**

Universal TB education and counseling (TEC) is routinely recommended for promoting knowledge and medication adherence, but the quality of delivery often varies because of inadequate clinic space, time, and health worker training. Peer-led counseling is a promising but understudied solution to these challenges. We sought to evaluate the efficacy of a peer-led TEC strategy among newly diagnosed adults initiating TB treatment in Kampala, Uganda.

**Methods:**

We conducted a longitudinal, pre-post implementation study comparing the routine, healthcare-worker-led and peer-led strategies for delivery of TEC to consecutive adult persons with TB at a large, public primary-care clinic. Trained staff administered a standardized TB knowledge survey to all persons with TB immediately following TEC. We compared TB knowledge by type of TEC received using t-tests.

**Results:**

We enrolled 161 persons with TB, 80 who received conventional TEC from health workers between June and July 2018, and 81 who received peer-led TEC between August and November 2019. The proportions of women (28% vs. 31%, p = 0.64) and persons living with HIV (36% vs 30%, p = 0.37) were similar in the pre- and post-implementation periods. Peer-led TEC was associated with a more significant increase in disease-specific (difference +21%, 95% CI +18% to + 24%, p < 0.0001) and treatment-specific TB knowledge scores (difference +14%, 95% CI + 10% to + 18%, p< 0.0001) than routine healthcare worker-delivered TEC. All TB knowledge constructs were significantly higher for those in the post-implementation period than those in the pre-implementation period. Nine participants met our threshold for adequate knowledge (score ≥ 90%) for disease-specific TB knowledge in the pre-implementation period compared to 63 (78%) in the post-implementation period (+67%, 95% CI + 55% − +78%, p < 0.001). Twenty-eight (35%) met the adequate knowledge threshold for TB treatment-specific knowledge in the pre-implementation period compared to 60 (74%) in the post-implementation period (+ 39%, 95% CI + 25 to + 53%, p < 0.0001). Finally, the proportion achieving TB treatment success (cure or completed) increased substantially from the pre-implementation period (n = 49, 68%) to the post-implementation period (n = 63, 88%), a difference of + 19% (95% CI + 6% to + 33%, p = 0.005).

**Conclusion:**

Our findings suggest that peer-led TEC is more efficacious than routine TEC at improving TB knowledge and treatment outcomes. Future studies should evaluate the implementation and effectiveness of the peer-led TEC strategy when scaled to a larger number of clinics.

## INTRODUCTION

Tuberculosis (TB) remains one of the leading causes of morbidity and mortality from a single infectious pathogen worldwide (1). Although the majority of those who develop TB can be cured with timely diagnosis and proper adherence to treatment (1), stigma (2) and inadequate TB knowledge (3, 4) are major barriers to successful treatment outcomes. Novel approaches are needed to deliver TB education and counseling (TEC) as they may help persons with TB (PWTB) better understand TB transmission, treatment, and prevention; empower them to disclose their own TB diagnoses to close contacts using accurate information (5); and in turn improve TB outcomes (6).

In many high TB-burden countries, TB treatment outcomes remain far below WHO’s target of ≥ 90% persons with TB achieving treatment success (1). One of the strongest predictors of treatment failure is inadequate baseline knowledge (7). Current TB treatment guidelines from the World Health Organization recommend that all PWTB routinely receive TEC at treatment initiation, although who should provide TEC and what topics should be covered during TEC are not specified (8). In a previous observational cohort study of PWTB with and without HIV initiating TB treatment in Uganda, we showed that baseline TB knowledge was low and, while routine TEC did improve TB knowledge, a substantial proportion of PWTB remained with an inadequate level of knowledge after TEC (9). Furthermore, these knowledge gaps were associated with decreased adherence to TB medications. In a concurrent qualitative study exploring TEC knowledge, attitudes, and practices with PWTB and healthcare workers in the same setting, we further discovered that a lack of space, time, and human resources; a lack of standardized content and integrated workflows for TEC; and high levels of stigma were all additional barriers to delivery of high-quality TEC (5). Staff involved in providing TB care at the clinic saw task-shifting of TEC to peer educators as one potential strategy to bridge these gaps, in emulation of a model used to support individuals starting antiretroviral therapy in HIV clinics.

Several studies have shown that peer educators can improve patient knowledge of and attitudes toward HIV and other infectious diseases by standardizing education (10), shifting most education and counseling activities away from overburdened healthcare workers (11, 12), and improving trust between people and health systems (13). However, there is limited data on the use of peer educators to support people with TB. Therefore, we sought to evaluate the efficacy of a peer-led TEC strategy for increasing TB knowledge in Kampala, Uganda.

## METHODS

### Study Design and Setting

We prospectively enrolled two cohorts of adult (age ≥ 18 years) PWTB in a pre-post implementation study to compare the efficacy of peer-led TEC to conventional TEC, as previously described (9). Using identical screening and recruitment methods in two consecutive years, we enrolled the two cohorts at Kisenyi Level IV Health Centre, a public primary care clinic located in a high-density, low-income urban neighborhood of Kampala and managed and operated by the Kampala Capital City Authority (KCCA). At Kisenyi and other government health facilities in Uganda, all TB evaluation and treatment services are offered free in a dedicated TB unit overseen by the Uganda National TB and Leprosy Programme (6). Kisenyi Health Centre diagnoses and treats > 750 PWTB annually, 40% of whom are persons living with HIV (PLHIV).

### Intervention Design

In April 2019, after completing formative research activities, including surveys and interviews with persons with TB, healthcare workers, and administrators, we designed the Theory-informed Education and Adherence Counseling to Improve HIV-TB Treatment Outcomes (TEACH) strategy. The TEACH strategy sought to improve the delivery of TEC by 1) standardizing educational delivery practices using a prompt card, 2) shifting responsibility for the delivery of TEC to peer educators, and 3) customizing individual treatment plans to anticipate and avoid barriers to medication adherence and retention in care (Appendix 1). A research team (IA, JG, EO) recruited peer educators from a pool of current and former PWTB recommended by clinic staff; all were ≥ 18 years old and had completed ≥ 2 months of TB treatment before recruitment. In ten half-day didactic and participatory sessions over two weeks, the research team trained peer educators to deliver TEC and verified their readiness to deliver TEC to real PWTB through mock TEC sessions observed by the research team. Peer educators also participated in monthly refresher training throughout the post-implementation period. Training sessions included lectures, role plays, and question-and-answer sessions designed to promote engagement and assess understanding.

### Study Eligibility

Participants were eligible for inclusion in the pre- or post-intervention cohort if they were (1) ≥ 18 years of age and (2) diagnosed with drug-susceptible TB of any type (microbiologically confirmed, extrapulmonary, or clinically diagnosed). We excluded those who (1) initiated treatment outside regular clinic hours (Monday-Friday, 8 am–3 pm), (3) had transferred into the study health center from another facility, or (4) declined to provide informed consent or could not do so.

### Measurements

A trained research assistant (L.N.) collected and recorded demographic and clinical characteristics using a written questionnaire. We used a previously developed TB knowledge survey (Appendix 2) to assess essential TB disease- and treatment-specific knowledge highlighted in clinical practice guidelines (14) and existing TB literacy materials (15). TB knowledge was collected after TEC delivery using password-protected electronic tablets equipped with commercial mobile survey software (Qualtrics, Seattle, WA) connected to a secure, cloud-based server.

### Statistical Analysis

We calculated scores for TB knowledge measures at the domain, construct, and question levels, averaging correct responses to questions within the same construct and normalizing each to a 100-point scale. We specified a minimum overall score > 90% as an adequate level of TB knowledge for each domain and construct, consistent with the Patient’s Charter for Tuberculosis Care statement that effective TB education is a fundamental right for all persons with TB (16).

We summarized baseline characteristics using descriptive statistics and compared differences in TB knowledge scores by cohort. To assess TEC efficacy, we constructed linear regression models comparing the TEC scores at the construct level by cohort period. STATA 18 (Stata Corporation, College Station, Texas) was used for all analyses. All statistical tests were conducted at a significance level of p < 0.05.

### Human Subjects Protection

Prior to recruiting each cohort, the study protocol was approved by the Makerere School of Public Health Higher Degrees Research Ethics Committee, the Uganda National Council for Science and Technology, and the Yale University Human Investigation Committee. All participants provided verbal informed consent. The investigators did not prospectively enter the study into a trials registry, but registered it with the Pan African Clinical Trials Registry retrospectively (PACTR# pending) as soon as they recognized that it met the definition of a clinical trial.

## RESULTS

**Figure 1:**
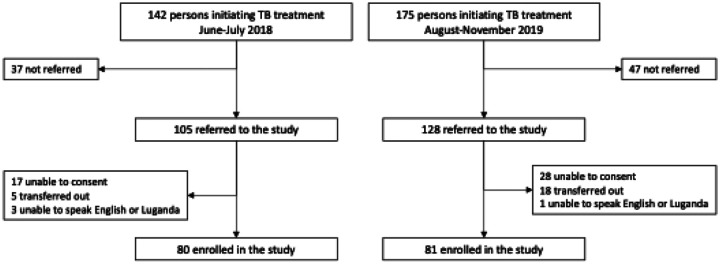
Study Enrollment Diagram. **Abbreviations:** MDR, multi-drug resistant TB; TB, tuberculosis; TEC, TB education and counseling Study staff trained TB clinic staff members to refer consecutive adults with newly diagnosed tuberculosis for screening for eligibility and enrollment in a pre-post implementation study conducted between June and July 2018 (routine TEC period, left-side of figure) and between August and November 2019 (peer-led TEC period, right-side of figure).

Among the 142 persons newly diagnosed with TB during the pre-implementation period (June-July 2018), 105 were referred to the study, and 80 were enrolled after excluding three who did not speak English or Luganda, 17 who were unable to consent, and five who immediately transferred to a different health facility for follow-up care (Fig. 1).

Among 175 newly diagnosed PWTB in the post-implementation period (August-November 2019), 128 were referred to the study, 81 were enrolled after excluding one patient who did not speak English or Luganda, 28 were unable to consent, and 18 immediately transferred to a different health facility for follow-up care.

### Baseline Characteristics

Baseline demographic and clinical characteristics were generally similar between the two cohorts (Table 1). Overall, PWTB were young, with those in the pre-implementation period having a median age of 31 years (Inter-quartile range (IQR) 25–38) versus 28 years (IQR 25–37) in the post-implementation period. Forty-three (54%) had at least a secondary school education pre-implementation versus 31 (38%) post-implementation (p = 0.32). Most were currently employed (61 (76%) pre-implementation vs. 66 (81%) post-implementation, p = 0.85). The proportions with bacteriologically confirmed TB were similar (63 (78%) pre-implementation vs. 58 (73%) post-implementation, p = 0.37).

### Post TEC TB Knowledge Scores

Post-TEC knowledge assessments showed substantial increases in correct responses with peer-led TEC compared to almost all questions and all constructs (Table 2). Participants who received routine TEC had significantly lower scores than those who received peer-led TEC for the disease-specific knowledge domain (76% vs 97%, difference + 21 %, 95% CI +18% to + 24%, p < 0.0001), the treatment-specific knowledge domain (81% vs 95%, difference +14%, 95% CI + 10% to + 18%, p < 0.0001), and for all constructs within both of these domains (Table 3). The largest gains in knowledge within the disease-specific knowledge domain were in the TB transmission construct (69% vs 97%, difference+ 29%, 95% CI + 23% to + 34%, p < 0.0001) and the TB pathogenesis construct (65% vs. 93%, difference + 28%, 95% CI + 22% to + 34%, p < 0.0001). The biggest gain in knowledge within the treatment-specific knowledge domain was the treatment regimen construct (74% vs 96%, difference + 23%, 95% CI +18% to + 28%, p < 0.0001).

Nine (11%) persons with TB in the routine-TEC cohort met our threshold for adequate knowledge (score ≥ 90% correct) for the disease-specific domain, compared to 63 (78%) in the peer-led cohort (difference + 67%, 95% CI + 55% to + 78%, p < 0.001). Similarly, within the treatment-specific knowledge domain, 28 (35%) persons with TB met our threshold for adequate knowledge (score ≥ 90% correct) in the routine cohort compared to 60 (74%) in the peer-led cohort (difference + 39%, 95% CI + 25% to + 53%, p < 0.0001).

Seventy-two PWTB had recorded TB treatment outcomes in each cohort. The proportion achieving TB treatment success (cure or completed) increased substantially from the pre-implementation period (n = 49, 68%) to the post-implementation period (n = 63, 88%), a difference of + 19% (95% CI + 6% to + 33%, p = 0.005).

## DISCUSSION

WHO recommends TEC for all PWTB initiating treatment to promote adherence and treatment completion and thereby increase the proportion of those who achieve a durable cure of TB (17). In addition, TEC exemplified a person-centered approach to TB care, one of the pillars of the WHO END TB Strategy. Our study is one of the first to demonstrate that a peer-led strategy for the delivery of TEC is associated with more significant increases in TB knowledge than routine TEC among newly diagnosed PWTB. These gains were large and broadly observed concerning all aspects of knowledge about TB disease and TB treatment. Notably, about three-quarters of all PWTB achieved adequate TB knowledge after only one day of peer-led TEC, compared with only one in nine in the usual care period. Additionally, those receiving peer-led TEC were much more likely to achieve favorable TB treatment outcomes.

Our findings are consistent with a previously published randomized controlled trial that demonstrated the effectiveness of a peer-led TB education intervention among PLWH in Nigeria (10). A meta-analysis of peer-led education for persons living with HIV showed that peer strategies were significantly associated with an increase in HIV knowledge, especially knowledge about HIV transmission routes (12). We also found that peer-led counseling was slightly less effective at increasing knowledge about TB treatment than knowledge about TB disease and that PWTB in both cohorts were less likely to retain TB knowledge after the initial counseling session, as in other studies (18). Ultimately, our findings suggest that peer-led TEC is an effective strategy for increasing knowledge, comparable to results seen with peer-led HIV education (11).

Peer-led education has been widely used in HIV programs to improve patient well-being and outcomes. For example, a pre-post, nested experimental study was conducted in South Africa to examine the effectiveness of peer-led HIV/AIDS education among prison inmates (19). The study found that not only was knowledge improved among inmates, but also an increase in safer sex practices after peer-led education (19). One of the main facilitators of improved knowledge and attitude found in prior studies included perceived trust between persons with TB and peer educators, allowing for a more collaborative learning environment (10, 20). Here, we were able to take the successes seen in HIV programs and adapt the peer-educator model to a TB population. Future studies should focus on the implementation and scale-up of this intervention to ensure that each person with TB receives the education they deserve.

### Limitations

Our study also had some limitations. First, because of the pre-post design that we employed, unmeasured confounders, including time effects, could explain some or all of the outcome improvements that we identified. Second, losses to follow-up were higher in the post-implementation cohort, primarily because of movement restrictions imposed during the COVID-19 pandemic, limiting our ability to measure TB knowledge over time. Therefore, differences seen on the day of TEC may not be generalizable to knowledge retention over time. Finally, we had relatively small sample sizes for both cohorts, limiting the generalizability and the statistical power to detect small differences.

### Strengths

Our study had several strengths. First, to our knowledge, our study is one of the first to compare the impact of peer-led and conventional education on TB disease-specific and treatment-specific knowledge among adult patients with newly diagnosed TB in a real-world setting. Second, our context-specific, locally adapted, and low-cost TEC intervention utilized few external resources and required little time to train peers and deliver TEC. This factor may facilitate implementation feasibility in low-income settings.

### Conclusions

Our study suggested that a peer-led TEC intervention is likely more efficacious at increasing knowledge and treatment outcomes than routine TEC that healthcare workers deliver. Future studies should evaluate the implementation, effectiveness, and mechanisms by which peer-led TEC affects TB medication adherence, retention in care, and outcomes.

## Figures and Tables

**Table 1:** Characteristics of study participants by cohort

Characteristic*	Routine TEC	Peer-Led TEC	p-Value^†^
	n = 80	n = 81	
**Age,** years [IQR]	31 [25–38]	28 [25–37]	0.27
**Female sex**	22 (28)	25 (31)	0.64
**Occupation**			
*Self-employed*	36 (45)	38 (47)	0.85
*Formally employed*	27 (34)	24 (30)	
*Not employed*	17 (21)	19 (23)	
**Education**			
*No prior schooling*	5 (6)	3 (4)	0.32
*Primary school*	44 (55)	35 (43)	
*Secondary school*	25 (31)	35 (43)	
*Higher education*	6 (8)	8 (10)	
**Persons living with HIV**	29 (36)	24 (30)	0.37
**Previous TB diagnosis**	24 (30)	17 (21)	0.19
**TB diagnosis**			
*Bacteriologically confirmed*	58 (73)	63 (78)	0.37
*Clinically diagnosed*	17 (21)	17 (21)	
*Extrapulmonary*	4 (5)	1 (1)	

**Abbreviations:** IQR, interquartile range; TB, tuberculosis; TEC, tuberculosis education and counseling.

* n, % unless otherwise specified. Numbers may not sum to totals due to missing data, and column percentages may not sum to 100% due to rounding;

† p-value for analysis of variance Rank Sum test (continuous variables) or Chi-square test (categorical variables)

**Table 2:** Comparison of Post Counseling Question Knowledge Scores by study period

KNOWLEDGE CONSTRUCT	Routine TEC	Peer-Led TEC	p-Value^†^
Questions	(%)	(%)	
PATHOGENESIS	**65**	**93**	**<0.0001**
TB attacks the lungs*	96	100	0.08
TB can attack other parts of the body outside of the lungs*	68	100	<0.0001
Everyone who is exposed to TB germs does not become ill*	31	80	<0.0001
SYMPTOMS	**80**	**98**	**<0.0001**
A cough that does not go away for two weeks is a warning sign of TB*	96	100	0.08
A cough that goes away after a few days is a warning sign of TB*	93	99	0.05
Loss of weight is a warning sign of TB*	90	99	0.02
General weakness is a warning sign of TB*	64	99	<0.0001
Vomiting is not a warning sign of TB*	56	87	<0.0001
TRANSMISSION	**69**	**97**	**<0.0001**
TB can be spread through the air*	98	99	0.56
How are TB germs released?	86	99	0.002
If you breathe in TB germs, where do they settle and grow?	71	98	<0.0001
TB cannot be spread through food*	46	97	<0.0001
TB cannot be spread through drinking water*	41	93	<0.0001
TB-HIV INTERACTIONS	**85**	**98**	**<0.0001**
Can you have HIV only (without TB)?	99	100	0.32
Can you have TB only (without HIV)?	99	99	0.99
Unprotected sex cannot spread TB*	58	96	<0.0001
PREVENTION	**83**	**98**	**0.001**
How can you stop the spread of TB?	83	98	0.001
TREATMENT MECHANISM	**96**	**100**	**0.0001**
How often can TB be cured if treatment is started in time?	100	100	1.00
When do you take your TB medications?	99	99	0.99
If you stop treatment before the full course of therapy, your TB becomes harder to cure*	96	100	0.08
If you stop taking the TB medication before the treatment period is finished, what might happen?	96	100	0.08
When can you stop taking the TB medication?	89	100	0.002
TREATMENT REGIMEN	**74**	**96**	**<0.0001**
What do your TB medications look like?	91	100	0.08
If you have TB, how long do you take the medication?	96	100	0.006
What should you do if your TB medication gives you yellow or red eyes, too much vomiting, intense body rash, or issues with sight?	89	95	0.15
What should you do if your TB medication gives you joint pain?	56	96	<0.0001
Name two potential side effects of TB treatment	65	94	<0.0001
What should you do if your TB medication gives you nausea?	44	93	<0.0001
TREATMENT MONITORING	**75**	**88**	**0.002**
When should you come to the clinic for your next appointment?	95	90	0.24
After starting the medication, how long does it usually take to start feeling better?	54	85	<0.0001

**Abbreviations:** IQR, interquartile range; TB, tuberculosis; TEC, tuberculosis education and counseling.

* Question asked in a Yes/No format;

† p-value for independent t-test of difference in between-subject means

**Table 3:** Post TB education and counseling knowledge scores by study period.

Domain*	Routine TEC	Peer-Led TEC	Difference (%)	p-Value^†^
*Construct* *	(95% CI)	(95% CI)	(95% CI)	
**Disease-Specific Knowledge**	76 (73–79)	97 (95–98)	21 (18–24)	<0.0001
*TB Transmission*	69 (63–74)	97 (95–99)	29 (23–34)	<0.0001
*TB Pathogenesis*	65 (60–70)	93 (90–96)	28 (22–34)	<0.0001
*TB Symptoms*	80 (76–84)	97 (96–99)	17 (13–22)	<0.0001
*TB Prevention*	83 (74–91)	98 (94–100)	15 (6–24)	0.001
*TB-HIV Interactions*	85 (81–89)	98 (97–100)	13 (9–18)	<0.0001
**Treatment-Specific Knowledge**	81 (78–85)	95 (93–97)	14 (10–18)	<0.0001
*Treatment Regimen*	74 (69–78)	96 (94–98)	23 (18–28)	<0.0001
*Treatment Monitoring*	74 (68–81)	88 (82–93)	13 (5–21)	0.002
*Treatment Mechanism*	96 (94–98)	100 (99–100)	4 (2–6)	0.0001

**Abbreviations:** CI, confidence intervals; TB, tuberculosis; TEC, tuberculosis education and counseling.

* Proportion of knowledge questions answered correctly;

† p-value for two-sided independent t-test of difference in means
